# Supplementary material for: Novel 1,8-Naphthalimide Derivatives Inhibit Growth and Induce Apoptosis in Human Glioblastoma
Source: Int J Mol Sci. 2024 Oct 29;25(21):11593. doi: 10.3390/ijms252111593 (PMC11546702; doi:10.3390/ijms252111593)
Supplement: Supplementary file 1 [file ijms-25-11593-s001.zip › ijms-3258477-supplementary.pdf]

(1) Supplementary Figure S1

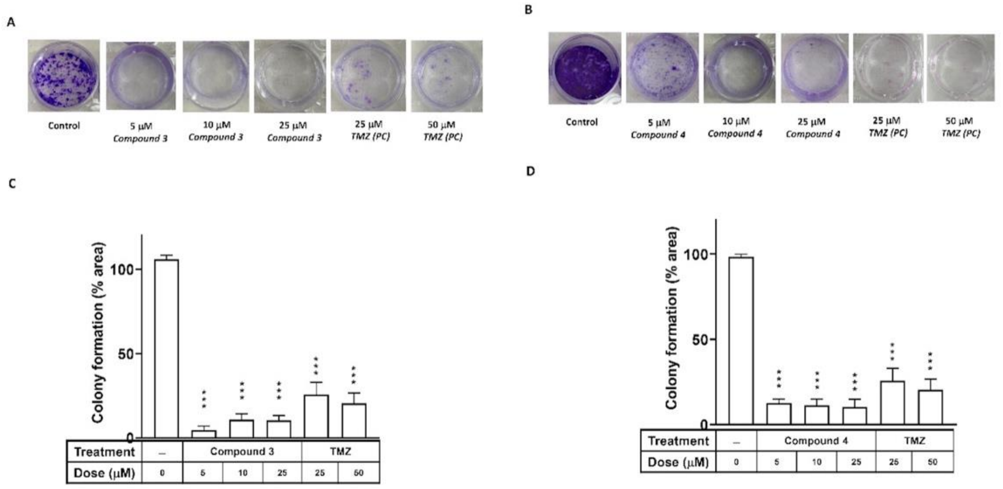

Supplementary Figure S1: Effects of compounds **3** and **4** on clonogenic survival of DBTRG-05MG GBM cells. DBTRG-05MG cells were seeded into culture plates and treated with compound **3** (**A**) or compound **4** (**B**) at a dose of 5, 10, or 25 μM, with TMZ used as a positive control (25 or 50 μM), for 8 days. Subsequently, the cells were fixed with 1% formalin containing 1% crystal violet, and colony formation was assessed using an inverted microscope. (**C**,**D**) Colony numbers were quantified in culture plates. Data are presented as the mean ± SE of three independent experiments, and statistical analysis revealed significant differences (*** $p < 0.001$) compared with the control group.

(2) Supplementary Figure S2

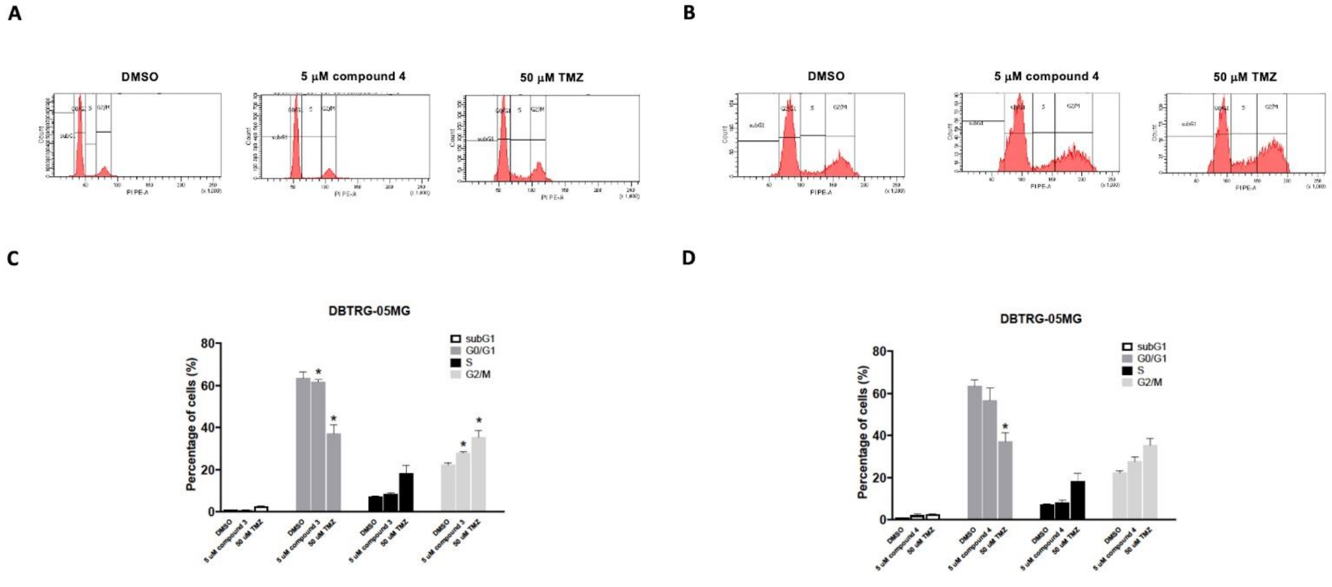

Supplementary Figure S2: Effects of compounds **3** and **4** on the cell cycle distribution of DBTRG-05MG GBM cells in vitro. DBTRG-05MG GBM cells were treated with 5 μM of compound **3** (**A**), 5 μM of compound **4** (**B**), or 50 μM of TMZ as a positive control. After 24 h, the cells were harvested and stained with propidium iodide (PI). The percentage distribution of cells in the sub-G1, G0/G1, S, and G2/M phases was analyzed through flow cytometry

(**C**,**D**). Data are presented as the mean ± SE of three independent experiments. Statistical analysis revealed significant differences (* $p < 0.05$) compared with the control group.

(3) Supplementary Figure S3

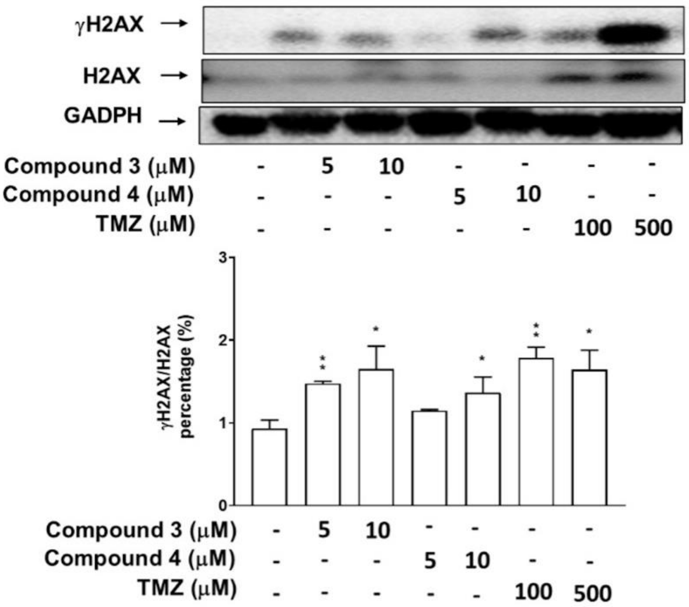

Supplementary Figure S3: Effects of compounds **3** and **4** on phosphorylation of H2AX (DNA damage marker) in DBTRG-05MG GBM cells. The expression of H2AX and its phosphorylation status at Ser 139 (γ-H2AX) were analyzed through immunoblotting by using antibodies against the phosphorylated and total protein, with GADPH serving as the loading control. Data are presented as means ± SEs (n = 3). * $p < 0.05$ compared with DMSO.

(4) Supplementary Figure S4

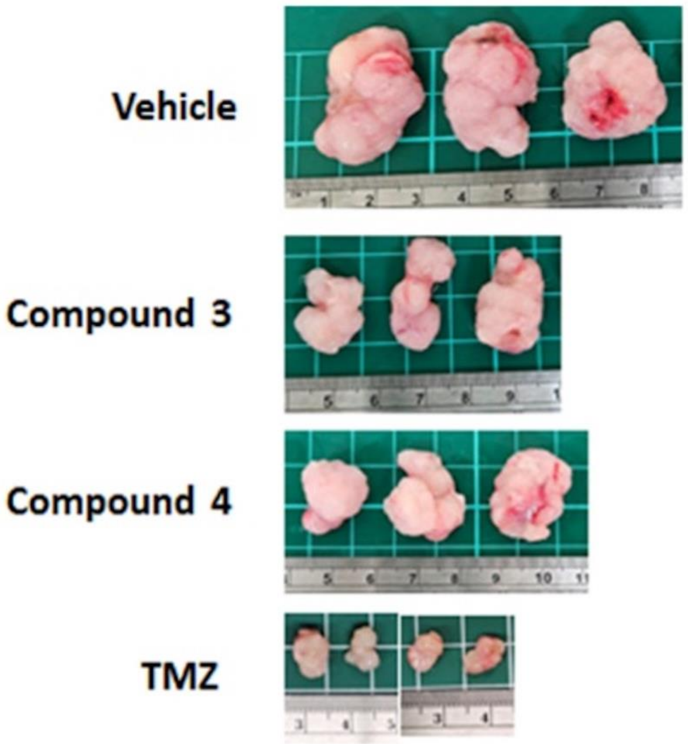

Supplementary Figure S4: In vivo antitumor activity of compounds **3** and **4** against U87 GBM xenografts. *Nonobese diabetic (NOD)/severe combined immunodeficient (SCID)* mice were subcutaneously implanted with U87 GBM cells and intraperitoneally injected with the vehicle (0.1% DMSO in saline), compound **3** (5 mg/kg), compound **4** (5 mg/kg), or

TMZ (12.5 mg/kg) thrice weekly. Tumor growth and size were recorded after the mice were euthanized. Tumors treated with compounds **3, 4** and TMZ were significantly smaller than the control vehicle.

(5) The ¹H and ¹³C NMR spectroscopic data, as well as HRMS data, for compounds 3 and 4

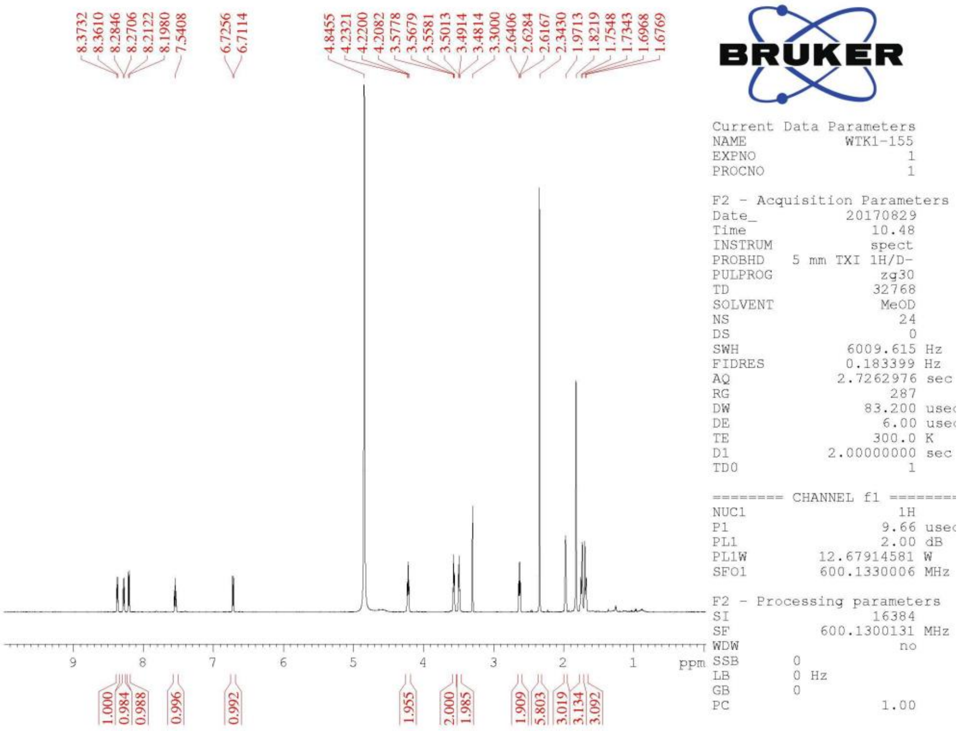

¹H NMR (600 MHz, $CDCl_3$) for compound **3**

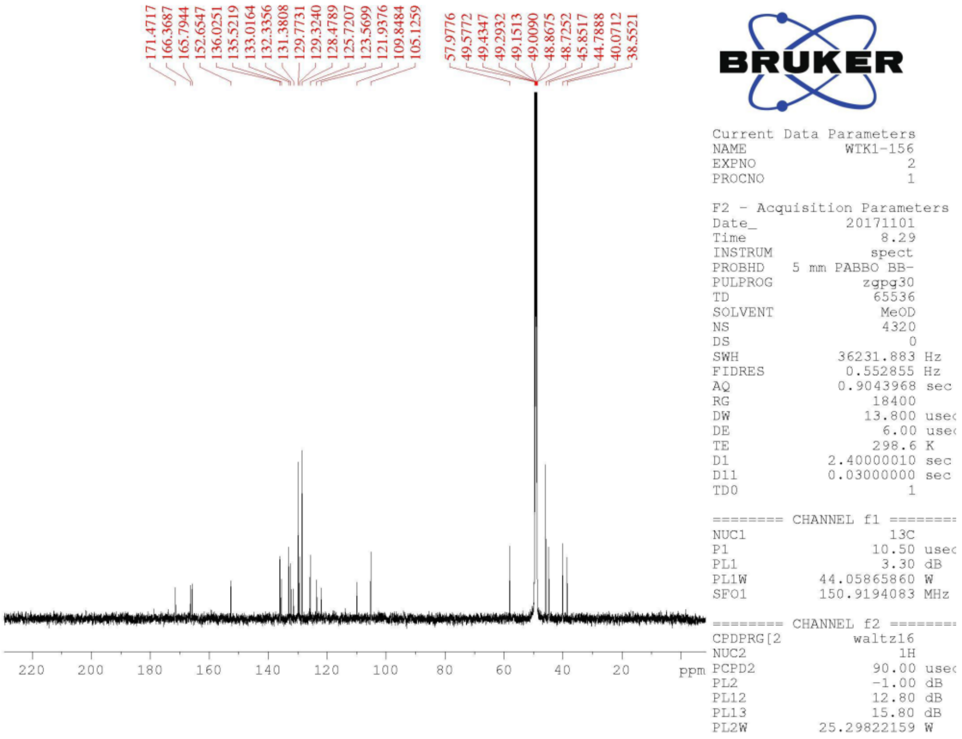

¹³C NMR (150 MHz, $CDCl_3$) for compound **3**

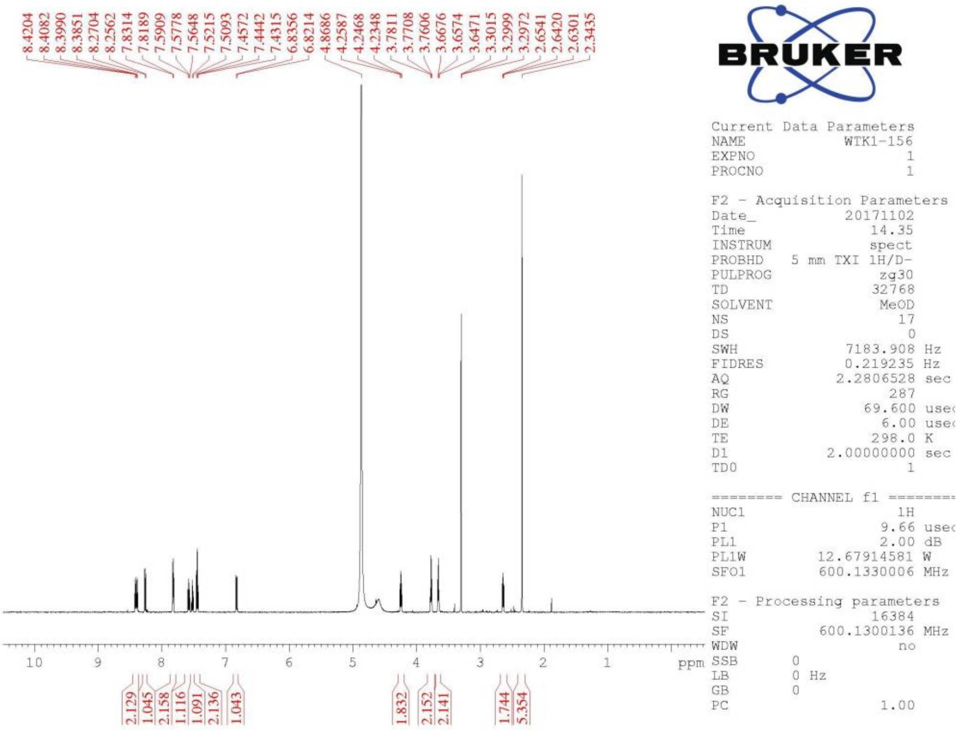

^{1}H NMR (600 MHz, $CDCl_3$) for compound **4**

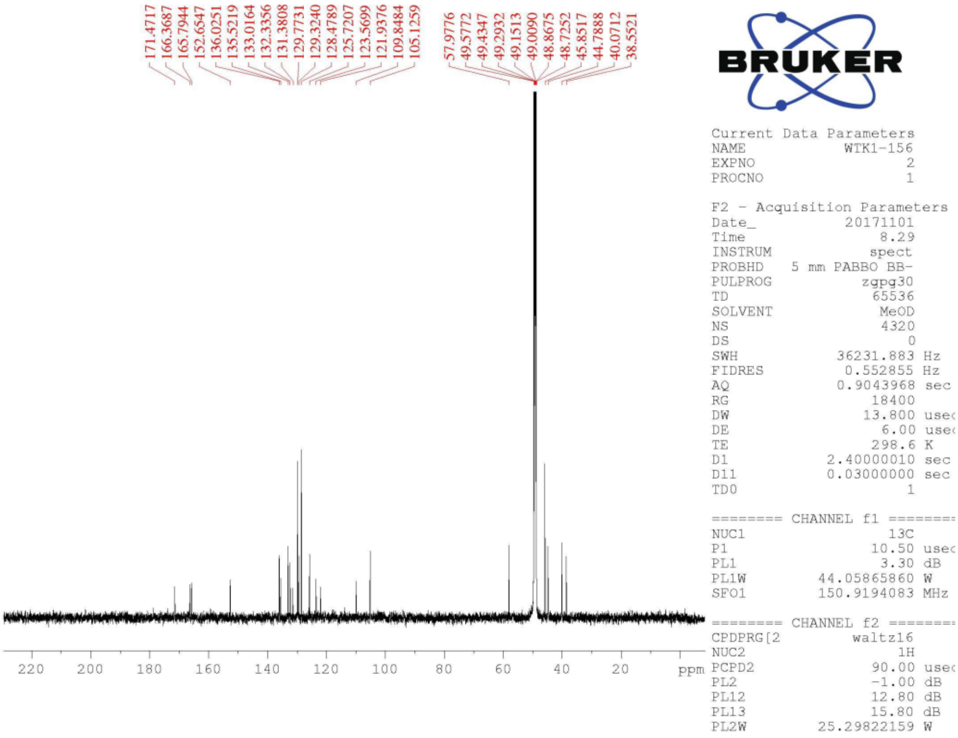

^{13}C NMR (150 MHz, $CDCl_3$) for compound **4**

(6) Synthetic Scheme and Procedure

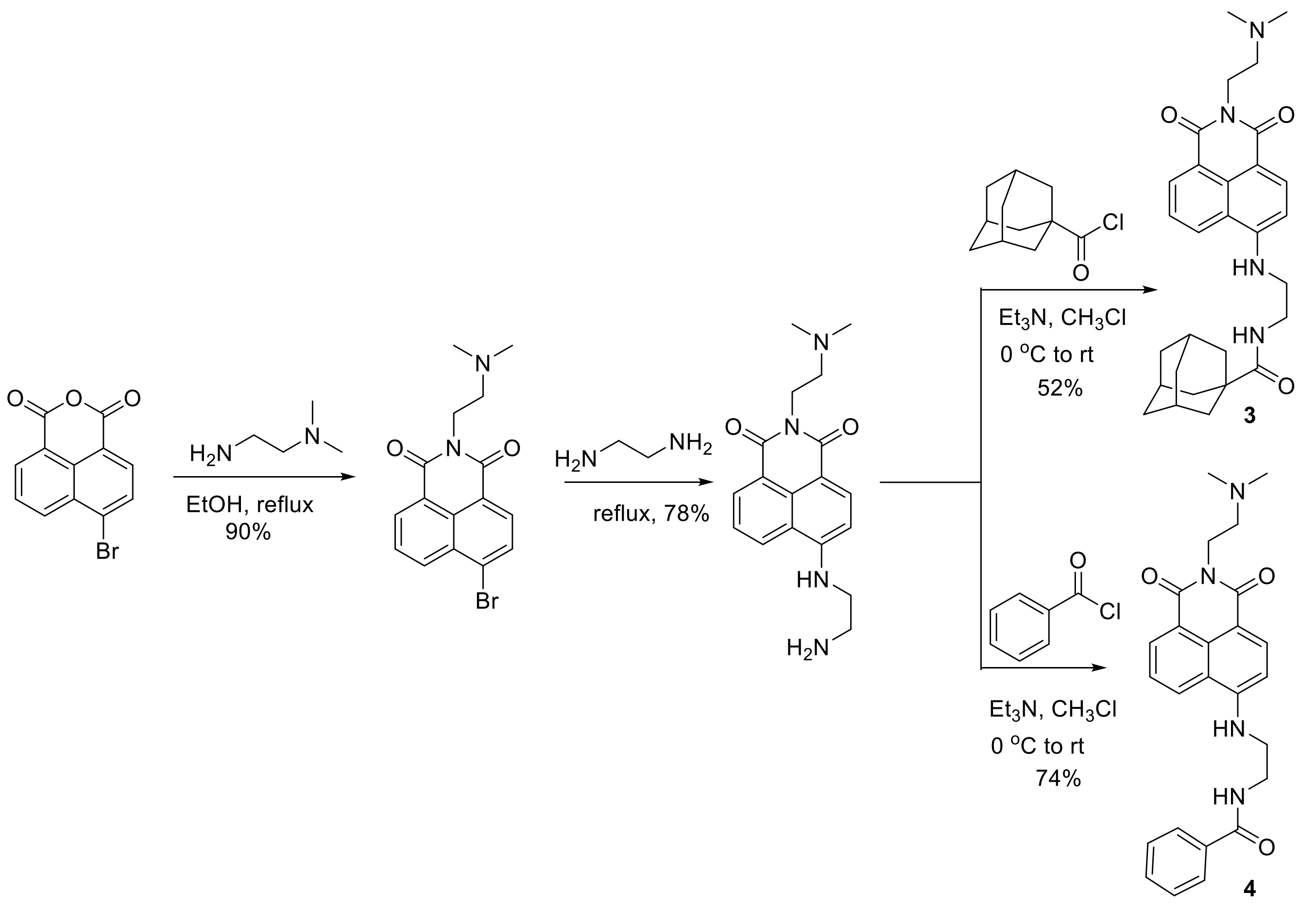

The synthetic procedure was previously reported by us (Tung, C.-H.; Lu, Y.-T.; Kao, W.-T.; Liu, J.-W.; Lai, Y.-H.; Jiang, S.-J.; Chen, H.-P.; Shih, T.-L. "Discovery of a more potent anti-cancer agent than C4-benzazole 1,8-naphthalimide derivatives against murine melanoma" *J. Chin. Chem. Soc.* **2020**, *67*, 1254-1262). We have provided the spectroscopic data on compounds **3** and **4** herein.

Compound **3**

Purification by flash column chromatography (230-400-mesh silica gel, CH_2Cl_2:EA = 4:1-1:1) yielded compound **3** as a yellow-orange solid (CH_2Cl_2:EA = 1:1, R_f = 0.5) at a 52% yield. Mp 174.8-176.3 ℃.^{1}H NMR (600 MHz, $CDCl_3$) δ 8.37 (d, J = 7.2 Hz, 1H), 8.28 (d, J = 8.4 Hz, 1H), 8.21 (d, J = 8.4 Hz, 1H), 7.55 (t, J = 7.8 Hz, 1H), 6.72 (d, J =9.0 Hz, 1H), 4.23 (t, J = 7.2 Hz, 2H), 3.57 (t, J = 6.0 Hz, 2H), 3.50 (t, J =6.0 Hz, 2H), 2.64 (t, J = 7.2 Hz, 2H), 2.34 (s, 6H), 1.97 (s, 3H), 1.75 (d, J = 12.6 Hz, 6H), 1.69 (t, J = 12.0 Hz, 6H). ^{13}C NMR (150 MHz, $CDCl_3$) δ 182.3, 166.2, 165.6, 152.6, 135.9, 132.2, 131.2, 129.2, 125.6, 123.4,121.7, 109.6, 105.0, 57.9, 45.8, 45.0, 42.0, 40.3, 39.5, 38.5, 37.7, 29.7.
HRMS (ESI) calculated for $C_{25}H_{36}N_4O_3[M+H]^+$: 489.2866. Value found: 489.2870.

Compound **4**

Purification by flash column chromatography (230-400-mesh silica gel, CH_2Cl_2:EA = 4:1-1:1) yielded compound **3** as an orange solid (CH_2Cl_2:EA = 1:1, R_f = 0.5) at a 74% yield. Mp 182-184 ℃. ^{1}H NMR (600 MHz, $CDCl_3$) δ 8.42 (d, J = 7.2 Hz,1H), 8.39 (d, J = 9.6 Hz, 1H), 8.27 (d, J = 8.4 Hz, 1H), 7.83 (d, J = 7.2 Hz, 1H), 7.59 (t, J = 8.4 Hz, 1H), 7.52 (t, J = 7.2 Hz, 1H), 7.45 (t, J = 7.8Hz, 1H), 6.83 (d, J = 9.0 Hz, 1H), 4.25 (t, J = 7.2 Hz, 2H), 3.78 (t, J = 6.0 Hz, 2H), 3.66 (t, J = 6.0 Hz, 2H), 2.65 (t, J = 7.2 Hz, 2H), 2.34 (s, 3H).^{13}C NMR (150 MHz, $CDCl_3$) δ 171.4, 166.3, 165.7, 152.6, 136.0, 135.5, 133.0, 132.3, 131.3, 129.7, 129.3, 128.4, 125.7, 123.5, 121.9, 109.8, 105.1, 57.9, 45.8, 44.7, 40.7, 38.5. HRMS (ESI) calculated for $C_{25}H_{26}N_4O_3[M+H]^+$: 431.2083. Value found: 431.2079.
